# Clinical value of serum tumor markers in assessing the efficacy of neoadjuvant chemotherapy in advanced ovarian cancer: single-center prospective clinical study

**DOI:** 10.3389/fonc.2024.1399502

**Published:** 2024-05-28

**Authors:** Jing Huang, Danyi Du, Hailong Chen, Deping Luo, Qi Wang, Chan Li, Yuanxiang Li, Ying Yu

**Affiliations:** ^1^ Department of Gynecology and Oncology, Ganzhou Cancer Hospital, Ganzhou, Jiangxi, China; ^2^ Shenzhen Hospital, Southern Medical University, Shenzhen, Guangdong, China; ^3^ Department of Clinical laboratory, Ganzhou Cancer Hospital, Ganzhou, Jiangxi, China

**Keywords:** ovarian cancer, neoadjuvant chemotherapy, human epididymal protein 4, efficacy of chemotherapy, predictive indicators

## Abstract

**Objective:**

This study aimed to assess the clinical importance of various biomarkers, including NLR, CEA, CA199, CA125, CA153, and HE4, through dynamic testing to evaluate the effectiveness of neoadjuvant chemotherapy (NACT) for individuals facing advanced ovarian cancer. This provides valuable information for tailoring treatment plans to individual patients, thereby leading to a more personalized and effective management of individuals facing ovarian cancer.

**Methods:**

The levels of NLR, CA125, CA199, CEA, CA153, and HE4 were detected before chemotherapy and after 3 courses of chemotherapy. Patients were categorized into ineffective and effective groups according to the effectiveness of NACT. To evaluate the factors influencing NACT’s effectiveness in individuals facing advanced ovarian cancer, receiver operating characteristic (ROC) curves, predictive modeling, and multifactorial regression analysis were employed.

**Results:**

In the effective group, the patients’ age, maximum tumor diameter, and CEA and HE4 levels of the patients were significantly higher compared to those in the ineffective group (*P* <.05). Additionally, the difference in HE4 levels before and after treatment between the effective and ineffective groups was statistically significant (*P*<.05). Multifactorial analysis showed that age and maximum tumor diameter were independent risk factors impacting the effectiveness of NACT in individuals facing advanced ovarian cancer (*P*<.05). The ROC curve for predicting the effectiveness of NACT in individuals facing advanced ovarian cancer showed a sensitivity of 93.3% for NLR and a specificity of 92.3% for CA199. HE4 emerged as the most reliable predictor, demonstrating a specificity of 84.6% and a sensitivity of 75.3%. The area under the curve of the combined CA125 and HE4 assays for predicting the ineffectiveness of NACT in individuals facing advanced ovarian cancer was 0.825, showcasing a specificity of 74.2% and a sensitivity of 84.6%.

**Conclusion:**

The predictive capacity for the effectiveness of NACT in individuals facing advanced ovarian cancer is notably high when considering the sensitivity of NLR and the specificity of CA199. Additionally, the combination of CA125 and HE4 assays can obtain a better predictive effect, which can accurately select patients suitable for NACT, determine the appropriate timing of the interval debulking surgery (IDS) surgery, and achieve a satisfactory tumor reduction effect.

## Introduction

The incidence of ovarian cancer has been steadily increasing, leading to approximately 140,000 women worldwide succumbing to this disease annually (1). Ovarian cancer ranks has the highest mortality rate among malignant tumors affecting the female reproductive system, representing a significant threat to women’s health (2, 3). Due to the ovaries’ deep location in the pelvis, ovarian cancer typically presents insidiously and is often difficult to detect in its early stages. Consequently, about 70% of patients are diagnosed at an advanced stage (4, 5). For these advanced cases, although targeted therapies have been incorporated into the treatment strategy for first-diagnosed ovarian cancer, the conventional treatment approach involves primary debulking surgery (PDS) followed by postoperative adjuvant chemotherapy (6–8). Additionally, for patients with multiple high-risk preoperative factors and where achieving optimal surgical outcomes is challenging, the combination of preoperative neoadjuvant chemotherapy (NACT), interval debulking surgery (IDS), and postoperative adjuvant chemotherapy is being explored (9, 10).

The role of NACT in treating advanced ovarian cancer remains controversial. A meta-analysis encompassing 835 ovarian cancer cases suggested that NACT is linked to a poorer prognosis (11). However, the prospective randomized controlled study (EORTC 55971) by Vergote et al. suggested that NACT-IDS is considered noninferior to PDS in individuals facing stage IIIC-IV ovarian cancer and is associated with fewer perioperative complications. Furthermore, the CHORUS study confirmed similar overall survival rate between NACT-IDS and PDS, with better prognoses reported in individuals facing stage IV ovarian cancer (12, 13). Despite ongoing debates regarding the efficacy of NACT-IDS, it has proven to enhance the likelihood of significant tumor reduction and reduce perioperative complication risks. Therefore, NACT-IDS should be considered a viable alternative treatment for individuals with The International Federation of Gynecology and Obstetrics (FIGO) stage III-IV ovarian cancer when satisfactory tumor reduction is not feasible with PDS alone (14, 15).

The neutrophil/lymphocyte ratio (NLR) in peripheral blood is recognized as an indicator of the body’s inflammatory and immune responses, with several studies highlighting its correlation with the prognosis of individuals suffering from ovarian cancer (16, 17). Moreover, studies have revealed that serum tumor markers, including carbohydrate antigen 153 (CA153), carcinoembryonic antigen (CEA), carbohydrate antigen 199 (CA199), carbohydrate antigen 125 (CA125), and human epididymal protein 4 (HE4), are linked to ovarian cancer prognosis. However, the sensitivity and specificity of these single-marker tests often fall short of ideal standards (18–23). To enhance tumor reduction rate, minimize perioperative complications, decrease postoperative chemotherapy resistance, and accurately select patients suitable for NACT, this study utilizes dynamic testing of NLR and tumor markers including CA125, CA199, CEA, CA153, and HE4 before and after NACT. The ability to accurately predict NACT’s effectiveness in individuals facing advanced ovarian cancer is crucial for developing personalized and precise treatment plans, ultimately aiming to improve the quality of life for these individuals.

## Materials and methods

### Ethics statement

The current study received approval from the Ethical Committee of Ganzhou Cancer Hospital (2024001), and all participants provided written informed consent. The research was conducted adhering to the Declaration of Helsinki’s principles concerning studies involving human subjects. After being fully informed about the nature of the study, each patient gave their written informed consent to participate.

### Patient inclusion criteria

Patients selected for the study were required to meet the following inclusion criterion: (1) aged 20-70 years; (2) pathologically confirmed diagnosis of epithelial ovarian cancer in patients who were newly diagnosed and undergoing their initial therapeutic intervention; (3) classified as stage IIIC-IV according to the FIGO 2013 criteria; (4) a treatment plan formulated by a multidisciplinary team(MDT)that included NACT combined with IDS and postoperative adjuvant chemotherapy; (5) an Eastern US Oncology Collaborative physical fitness score <2 and a Karnofsky score ≥80; (6) no severe organic lesions in the liver, lungs, heart, or other important organs, and capable of tolerating chemotherapy and surgery; and (7) availability of complete clinical information and willingness to provide signed informed consent.

### Patient exclusion criteria

Patients were excluded from the study for the following reasons: (1) presence of a nonepithelial malignant tumor of the ovary; (2) staging as I-IIIB according to FIGO 2013 criteria; (3) presence of serious organic lesions in the liver, lungs, heart, and other important organs, rendering them unable to tolerate chemotherapy and surgery; (4) recurrent or uncontrolled ovarian cancer; (5) absence of a treatment plan discussion with MDT. 

### Clinical data

From January 2020 to December 2023, the gynecological oncology department at Ganzhou Cancer Hospital/Ganzhou Cancer Centre enrolled a total of 102 individuals facing advanced ovarian cancer. Participants ranged in age from 40 to 74 years, with a median age of 57.0 years, all of whom met the specified inclusion criteria (**Table 1**).

**Table 1:** General clinical data of the patients (n=102).

Parameter	Number of patients, n (%)
Age, years	
40~50	29(28.43)
51~60	47(46.08)
61~70	20(19.61)
>70	6(5.88)
Pathological type	
High-grade serous adenocarcinoma	85 (83.33)
Low grade serous adenocarcinoma	3(2.94)
Poorly differentiated adenocarcinoma	7(6.86)
Clear cell carcinoma	4(3.92)
Small cell malignant tumor	3(2.94)
Maximum tumor diameter	
>37mm	82(80.39)
≤ 37mm	20(19.61)
FIGO stage	
IIIC	54 (52.94)
IVA	12(11.76)
IVB	36(35.29)

### Methods of chemotherapy

NACT in this protocol involves the administration of a platinum-based regimen. This includes paclitaxel, dosed at 135-175 mg/m^2^, delivered via a slow intravenous drip over 3 hours, and cisplatin, dosed at 50-75 mg/m^2^, also administered intravenously. Prophylactic measures include the oral intake of 10 mg of dexamethasone and acid-suppressing drugs to protect the gastric mucosa, administered 6 hours before initiating the chemotherapy. Additionally, there is strict monitoring of respiration rate, blood pressure, pulse, heart rate, and other vital signs. NACT is administered daily for a 3-week period, totaling three courses of chemotherapy.

### Observation indicators

The tumor size, distant metastasis, and dynamic changes in serum tumor marker levels (NLR, CEA, CA199, CA153, CA125, and HE4) were observed and recorded before and after administering NACT.

### NACT efficacy determination criteria

According to the World Health Organization Response Evaluation Criteria in Solid Tumors (RECIST) version 1.1 (24), treatment efficacy was classified into four categories: progressive disease (PD), no change (NC), partial response (PR), and complete response (CR), with CR+PR as effective and PR+NC as ineffective.

### Statistical analysis

Statistical analyses were conducted utilizing SPSS (version 26.0; IBM Corp). Measurement data that adhered to a normal distribution were expressed as mean ± SD (μ±σ), and a *t*-test was carried out. Count data were expressed as the number of cases and percentage, and chi-square test was performed. To analyze the factors influencing the efficacy of NACT in individuals facing advanced ovarian cancer, Cox proportional hazards regression and logistic regression analyses were utilized. R (version 4.2.2; The R Project for Statistical Computing) software and *rms* software package were used to draw receiver operating characteristic (ROC) curve, calibration curve, and column line. Additionally, decision curve analysis (DCA) was utilized to assess the diagnostic efficacy of the prediction model. *P* values <.05 were considered statistically significant.

## Results

### Evaluating NACT efficacy

Of the 102 patients, 8 patients achieved CR, 81 patients achieved PR, 5 patients showed NC, and 8 patients showed PD after three chemotherapy sessions. Moreover, CR+PR was considered effective (n=89) and PR+NC was considered ineffective (n=13). The results are presented in **Table 2**.

**Table 2:** Efficacy of neoadjuvant chemotherapy/case (%) (n=102).

Parameter	Number of patients, n (%)
CR	8 (7.84)
PR	81 (79.41)
NC	5 (4.90)
PD	8 (7.84)

CR, complete response; PR, partial response; NC, no change; PD, progressive disease.

### Comparison of the basic conditions of patients in the two groups

There was no statistically significant difference between the effective and the ineffective groups in terms of FIGO staging of cancer, and the levels of tumor markers NLR, CA125, CA199, and CA153 (*P*>.05); however, there was a statistically significant difference in terms of age, pathological type, maximum diameter of tumor, and the levels of CEA and HE4 (*P*<.05), compared with the other groups (**Table 3**).

**Table 3:** Comparison of the basic conditions of patients in the two groups (n=102).

Parameter	Efficiently	Null	χ2/t	P
Age, years				
40~50	20	9	13.28	.004
51~60	43	4		
61~70	20	0		
>70	6	0		
Pathological type				
High-grade serous adenocarcinoma	76	9	14.224	.007
Low grade serous adenocarcinoma	3	0		
Poorly differentiated adenocarcinoma	3	4		
Clear cell carcinoma	4	0		
Small cell malignant tumor	3	0		
Maximum tumor diameter				
>37mm	78	4	23.27	<0.01
≤ 37mm	11	9		
FIGO stage				
IIIC	45	9	5.58	.061
IVA	9	3		
IVB	35	1		
NLR (4.42±3.04)				
>4.42	39	4	0.79	.373
≤ 4.42	50	9		
CA125(990.87±1673.62)				
>990.87	18	0	3.19	.074
≤ 990.87	71	13		
CA199(57.44±174.20)				
>57.44	14	1	0.58	.445
≤ 57.44	75	12		
CEA(2.07±3.69)				
>2.07	28	0	5.64	.018
≤ 2.07	61	13		
CA153(78.52±86.14)				
>78.52	29	3	0.48	.490
≤ 78.52	60	10		
HE4(422.95±490.93)				
>422.95	30	0	6.21	.013
≤ 422.95	59	13		

NLR, neutrophil-to-lymphocyte ratio; CA125, cancer antigen 125; CA199, carbohydrate antigen 19-9; CEA, carcinoembryonic antigen; CA153, cancer antigen 153; HE4, human epididymis protein 4.

### Comparison of NLR, CA125, CA199, CEA, CA153, and HE4 levels between the two groups of patients

The independent samples *t*-test revealed that the serum HE4 levels of the patients in the effective group before chemotherapy were higher than that in the ineffective group, and the NLR levels of the patients in the effective group after chemotherapy were higher than that in the ineffective group, and the difference was statistically significant (*P*<.05; **Table 4**). The difference in HE4 levels before and after the treatment between the effective and ineffective groups was statistically significant (*P* <.05; **Table 5**).

**Table 4:** Analysis of factors affecting the effectiveness of neoadjuvant chemotherapy for advanced ovarian cancer.

group		NLR	CA125	CA199	CEA	CA153	HE4
Active group	Before chemotherapy	4.36±2.80	1001.03±1762.51	62.85±185.48	2.19±3.93	82.29±89.7	470.96±507.9
	After chemotherapy	3.32±2.13	255.60±902.64	14.89±11.25	2.32±4.43	20.21±16.43	151.87±246.13
Invalid group	Before chemotherapy	4.84±4.46	236.68±297.49	20.39±37.66	1.24±0.43	52.69±51.15	94.21±50.57
	After chemotherapy	1.82±1.08	108.46±148.64	10.51±5.18	1.13±0.44	22.44±9.95	76.08±33.25
*t* value between groups before chemotherapy		-0.52	1.76	0.82	0.86	1.16	2.66
*p* value between groups before chemotherapy		0.603	0.082	0.414	0.391	0.249	0.009
*t* value between groups after chemotherapy		2.47	0.58	1.38	0.96	-0.48	1.10
*p* value between groups after chemotherapy		0.015	0.56	0.171	0.337	0.635	0.272

NLR, neutrophil-to-lymphocyte ratio; CA125, cancer antigen 125; CA199, carbohydrate antigen 19-9; CEA, carcinoembryonic antigen; CA153, cancer antigen 153; HE4, human epididymis protein 4.

**Table 5:** Comparison of the difference in NLR, CA125, CA199, CEA, CA153, HE4 before and after chemotherapy between the effective group and the ineffective group.

group	NLR	CA125	CA199	CEA	CA153	HE4
Active group	1.05±3.42	845.43±1541.96	47.96±178.43	-0.13±0.86	62.09±83.97	319.09±420.55
Invalid group	3.01±3.88	128.21±347.43	9.87±37.02	0.12±0.76	30.25±57.10	18.13±59.66
*t* values between groups before and after chemotherapy	-1.90	1.66	0.76	0.98	1.32	2.57
*P* values between groups before and after chemotherapy	0.06	0.099	0.447	0.328	0.19	0.012

NLR, neutrophil-to-lymphocyte ratio; CA125, cancer antigen 125; CA199, carbohydrate antigen 19-9; CEA, carcinoembryonic antigen; CA153, cancer antigen 153; HE4, human epididymis protein 4.

### Analysis of factors affecting the efficacy of NACT in patients with advanced ovarian cancer

Univariate Cox analysis showed that age, maximum tumor diameter, and HE4 levels were risk factors affecting the efficacy of NACT in patients with advanced ovarian cancer. Multifactorial analysis showed that age and maximum tumor diameter were independent risk factors affecting the efficacy of NACT in patients with advanced ovarian cancer (**Table 6**).

**Table 6:** Analysis of factors affecting the effectiveness of neoadjuvant chemotherapy for advanced ovarian cancer.

Variable	Single factor analysis			Multifactor analysis		
	HR	95%CI	P	HR	95%CI	P
Age	0.912	(0.852~0.977)	0.009	0.900	(0.823~0.985)	0.022
Pathological type	–	–	0.409	–	–	–
Maximum tumor diameter	0.98	(0.965~0.996)	0.014	0.983	(0.968~0.998)	0.024
FIGO stage	–	–	0.103	–	–	–
NLR	–	–	0.624	–	–	–
CA125	–	–	0.128	–	–	–
CA199	–	–	0.514	–	–	–
CEA	–	–	0.392	–	–	–
CA153	–	–	0.285	–	–	–
HE4	0.989	(0.980~0.999)	<0.01	–	–	0.084

NLR, neutrophil-to-lymphocyte ratio; CA125, cancer antigen 125; CA199, carbohydrate antigen 19-9; CEA, carcinoembryonic antigen; CA153, cancer antigen 153; HE4, human epididymis protein 4.

### Establishment of a clinical prediction model for NACT in patients with advanced ovarian cancer

**Figure 1:**
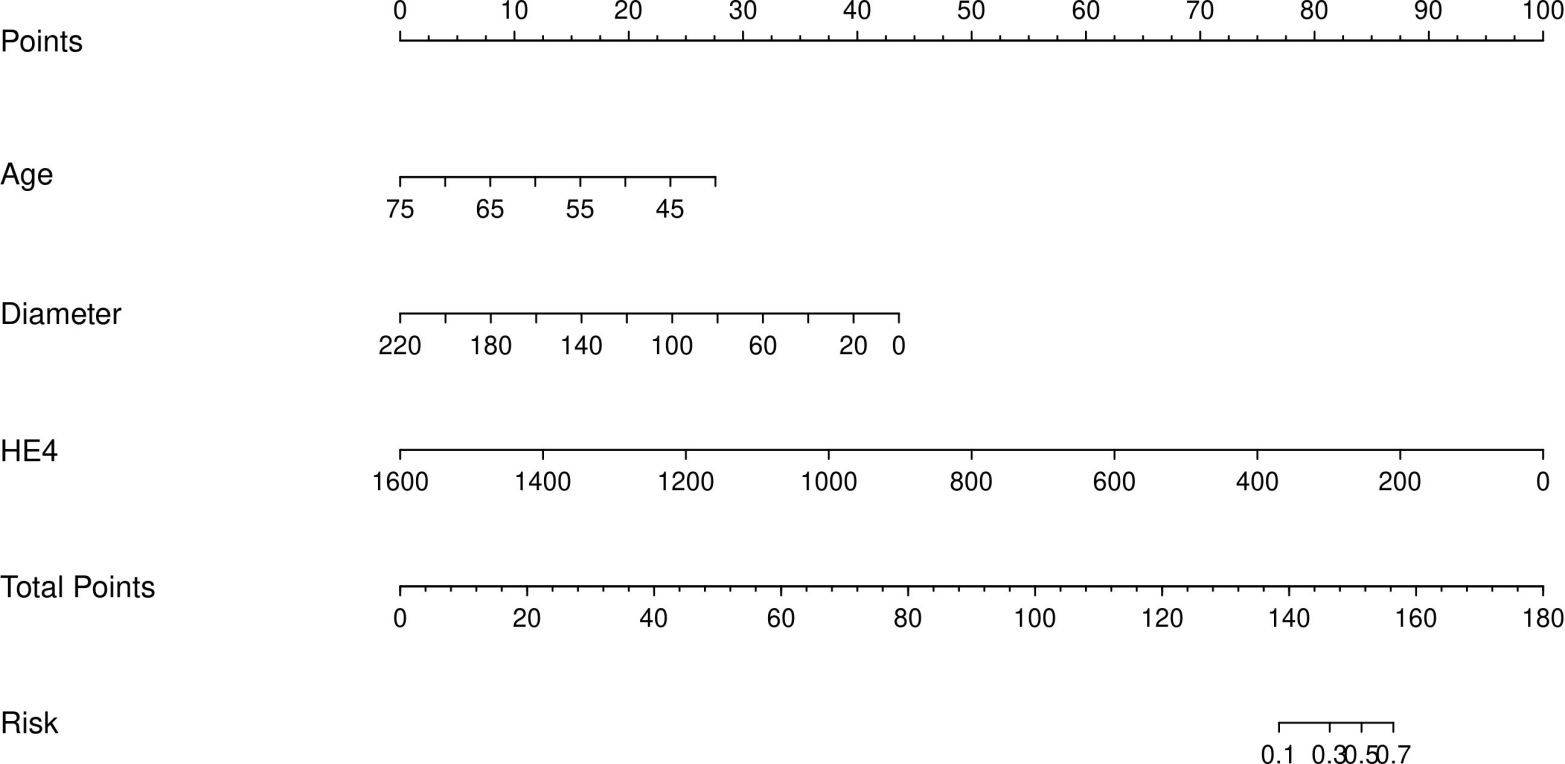
Neoadjuvant chemotherapy for advanced ovarian cancer predictive column line chart.

**Figure 2:**
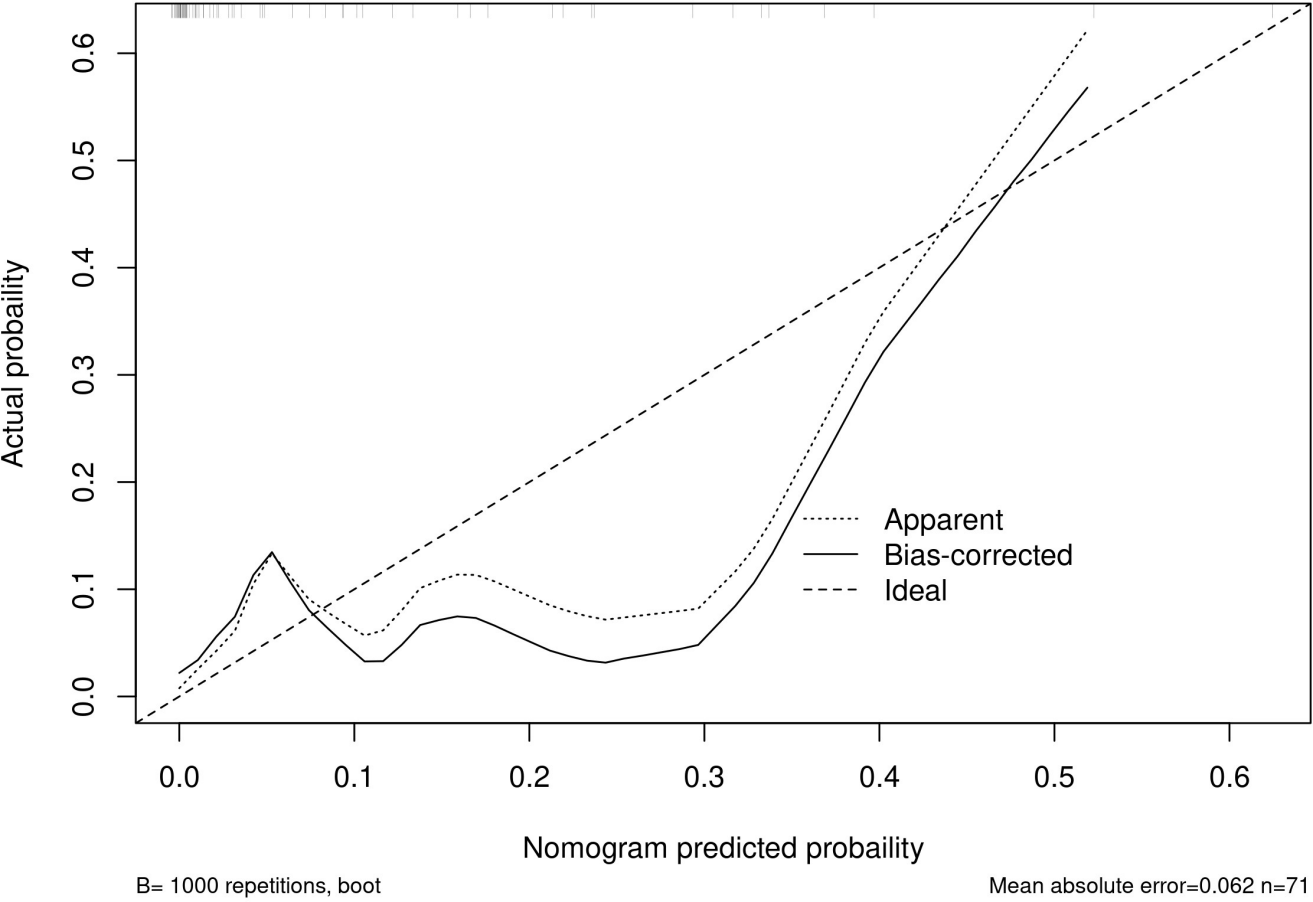
Training set calibration analysis.

**Figure 3:**
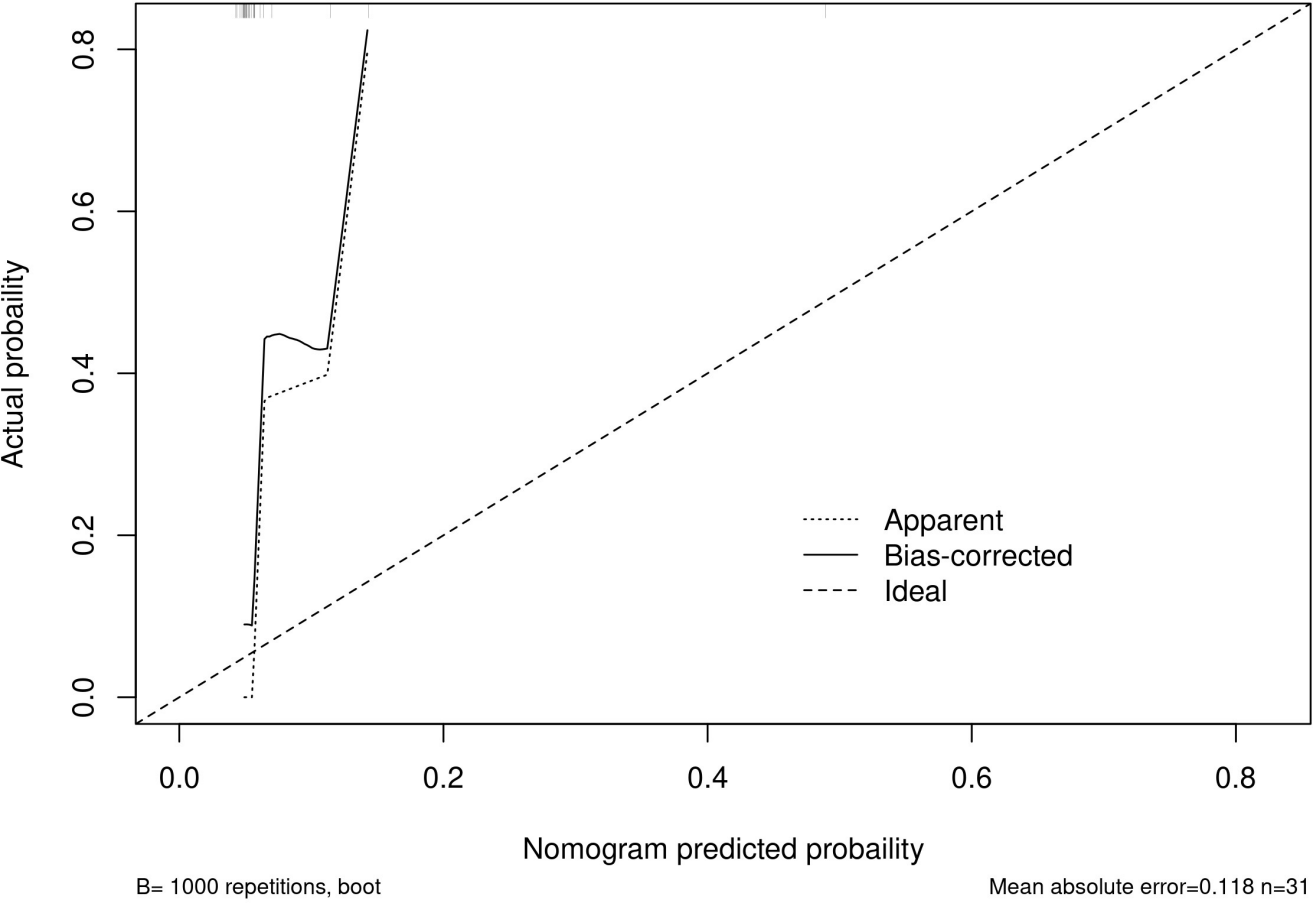
Validation set calibration analysis.

**Figure 4:**
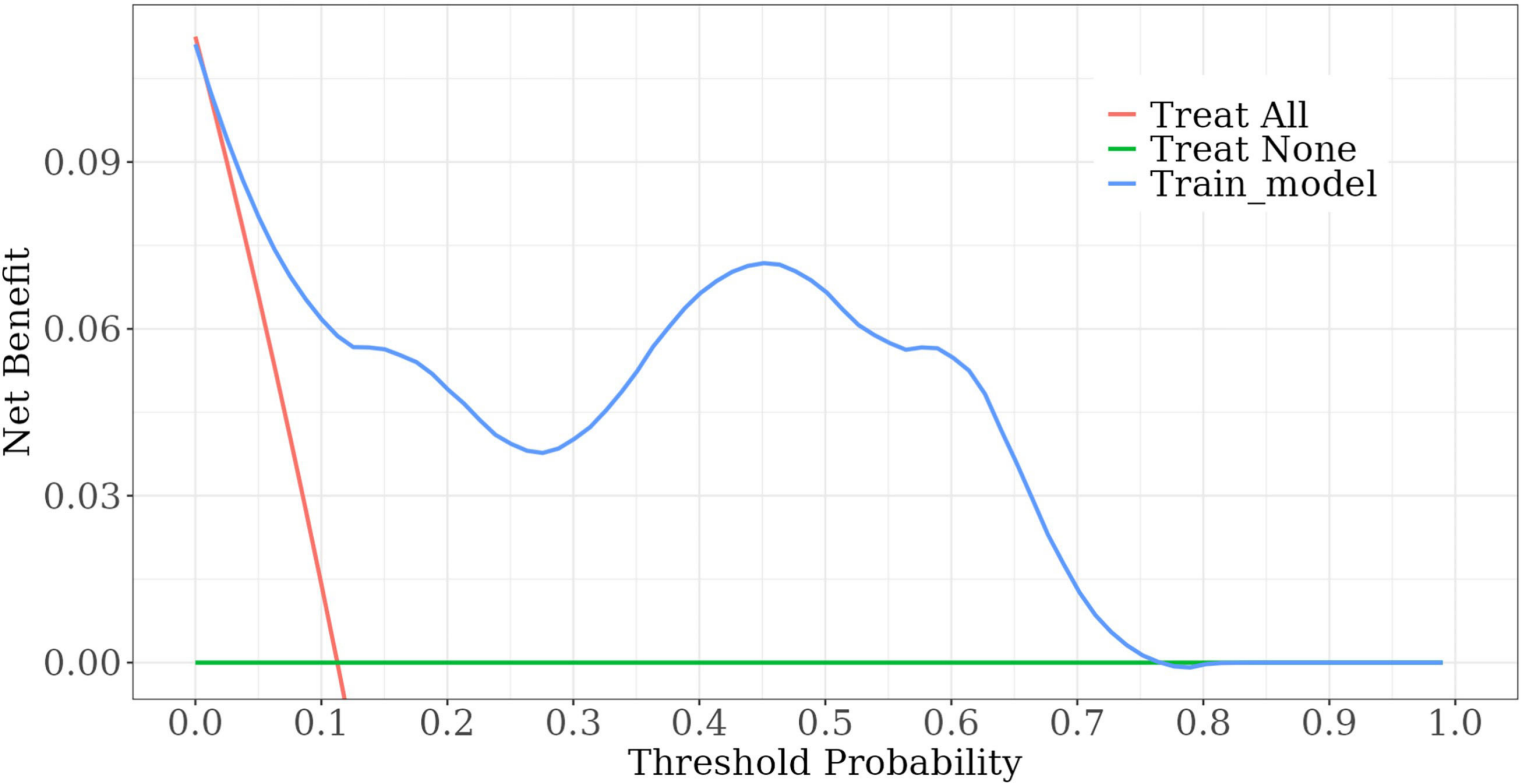
Validation set: decision curve analysis curve.

**Figure 5:**
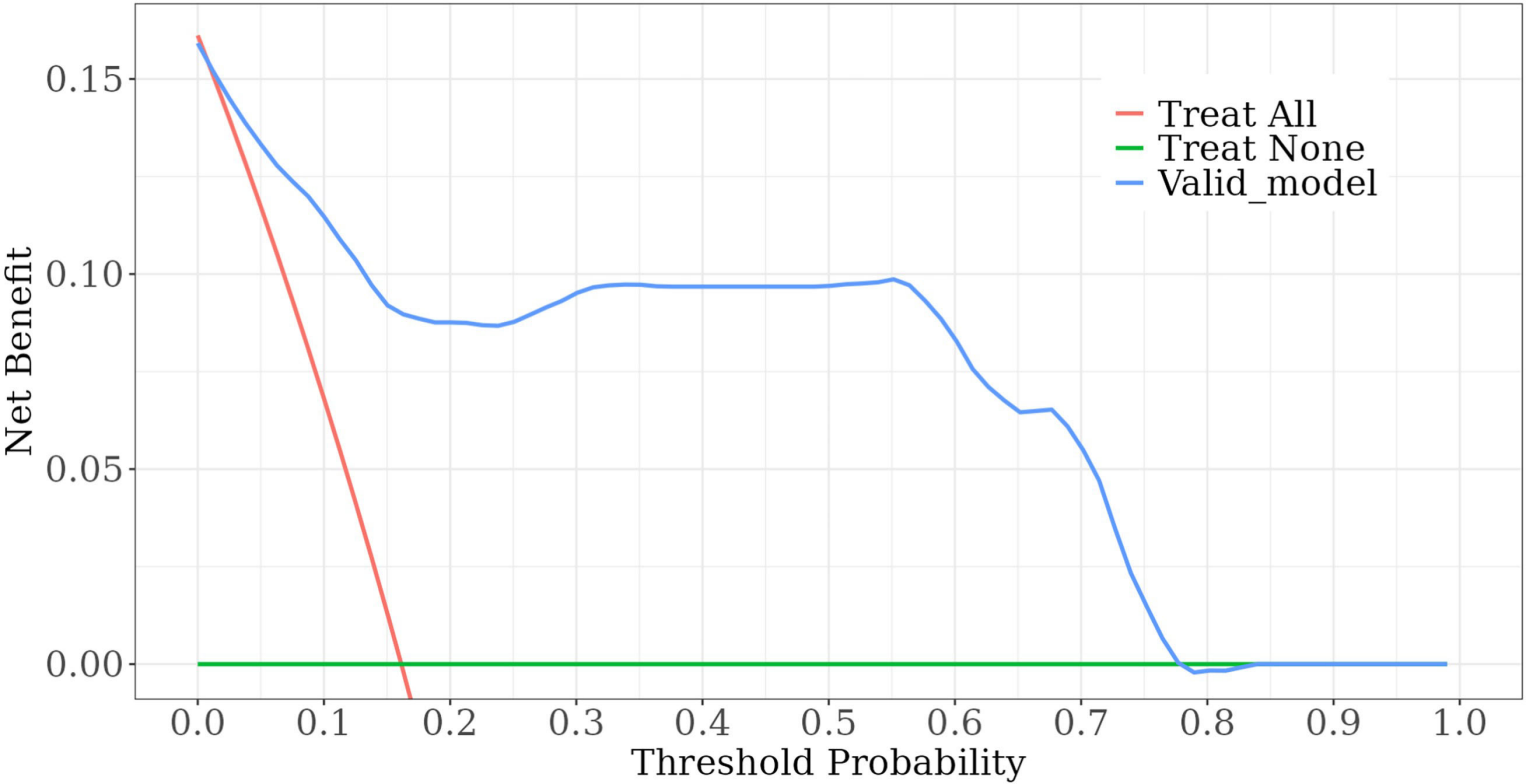
Training set: decision curve analysis curve.

**Figure 6:**
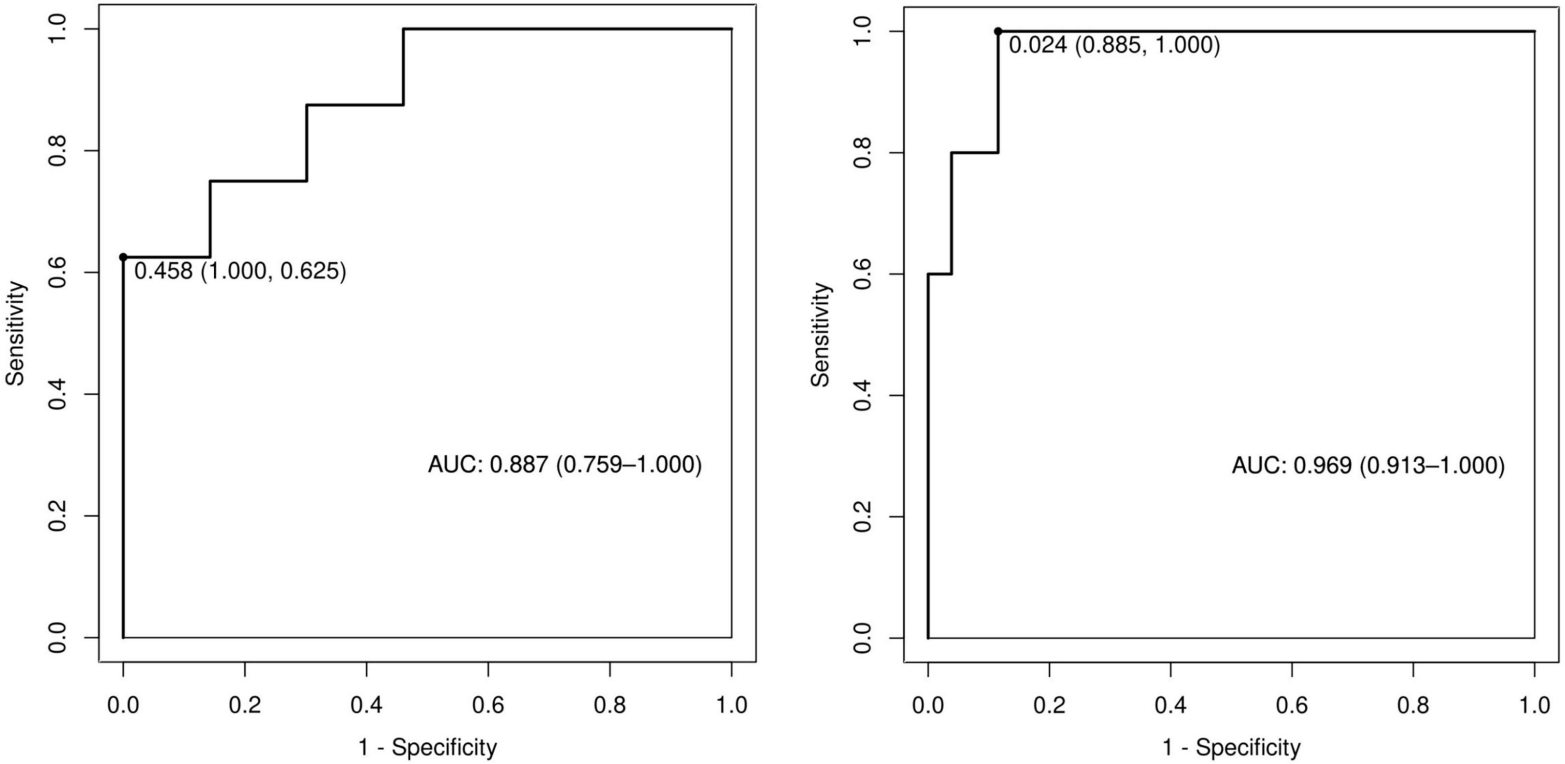
Receiver operating characteristic curves of predictive models in training and validation sets.

Among the independent risk factors, 70% of the data were randomly selected as the training set, and 30% were selected as the validation set to build a clinical prediction model for NACT in patients with advanced ovarian cancer. The column line graphs (**Figure 1**), training set versus validation set calibration curves (**Figures 2**, **3**), and clinical decision curves versus clinical impact curves (**Figures 4**, **5**) were plotted. The differentiation and calibration of the predictive model were validated by the area under the ROC curve and the Hosmer-Lemeshow test, respectively, and the area under the ROC curve of the validation set was higher than that of the training set (**Figure 6**).

### Predictive effects of detecting serum NLR, CA125, CA199, CEA, CA153, and HE4 levels on the efficacy of NACT in advanced ovarian cancer

**Figure 7:**
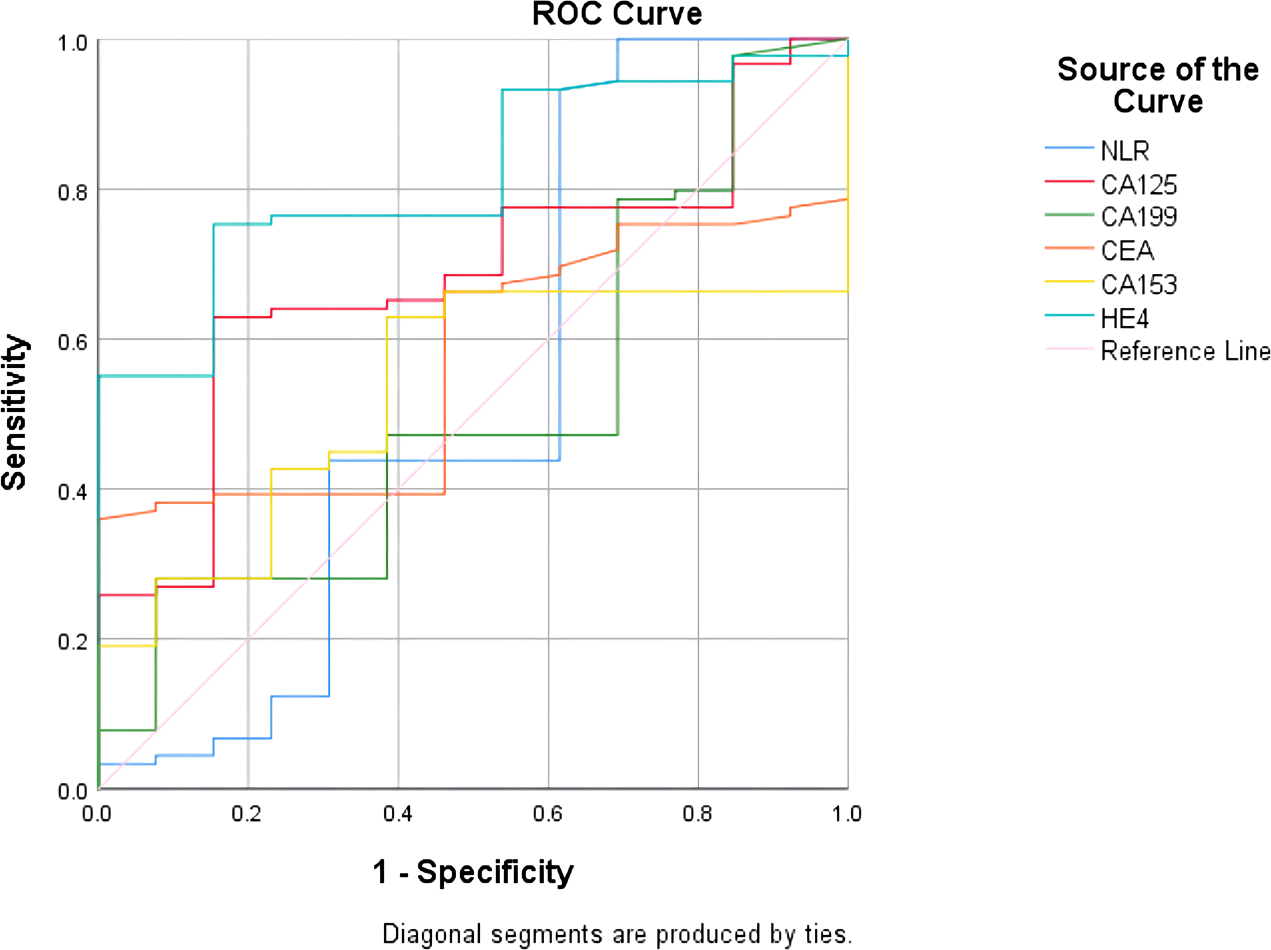
Receiver operating characteristic curves for each test to predict. efficacy of neoadjuvant chemotherapy in patients with ovarian cancer.

The ROC curve showed that NLR had the highest sensitivity (93.3%) in predicting the efficacy of NACT in patients with advanced ovarian cancer, CA199 had the highest specificity of 92.3% in predicting NACT efficacy in patients with advanced ovarian cancer, CA153 had sensitivity of 62.9% and a specificity of 61.5% for predicting NACT efficacy in patients with advanced ovarian cancer, and HE4 had a sensitivity of 75.3% and specificity of 84.6% in predicting the efficacy of NACT in patients with advanced ovarian cancer. HE4 was the best predictor of the efficacy of NACT in patients with advanced ovarian cancer (**Figure 7**; **Table 7**).

**Table 7:** Results of ROC curves for each test effective for neoadjuvant chemotherapy in ovarian cancer.

Variable	AUC (95% CI)	Cutoff value	Sensitivity	Specificity
NLR	0.535(95%CI 0.321~0.75)	1.20	93.3%	38.5%
CA125	0.68 (95%CI 0.546~0.814)	206.30	62.9%	37.1%
CA199	0.512(95%CI 0.346~0.678)	15.51	28.1%	92.3%
CEA	0.57 (95%CI 0.445~0.695)	1.85	36%	0%
CA153	0.531(95%CI 0.404~0.657)	27.16	62.9%	61.5%
HE4	0.817(95%CI 0.72~0.914)	99.79	75.3%	84.6%

NLR, neutrophil-to-lymphocyte ratio; CA125, cancer antigen 125; CA199, carbohydrate antigen 19-9; CEA, carcinoembryonic antigen; CA153, cancer antigen 153; HE4, human epididymis protein 4.

### Predictive effects of combined testing on NACT ineffectiveness in advanced ovarian cancer

**Figure 8:**
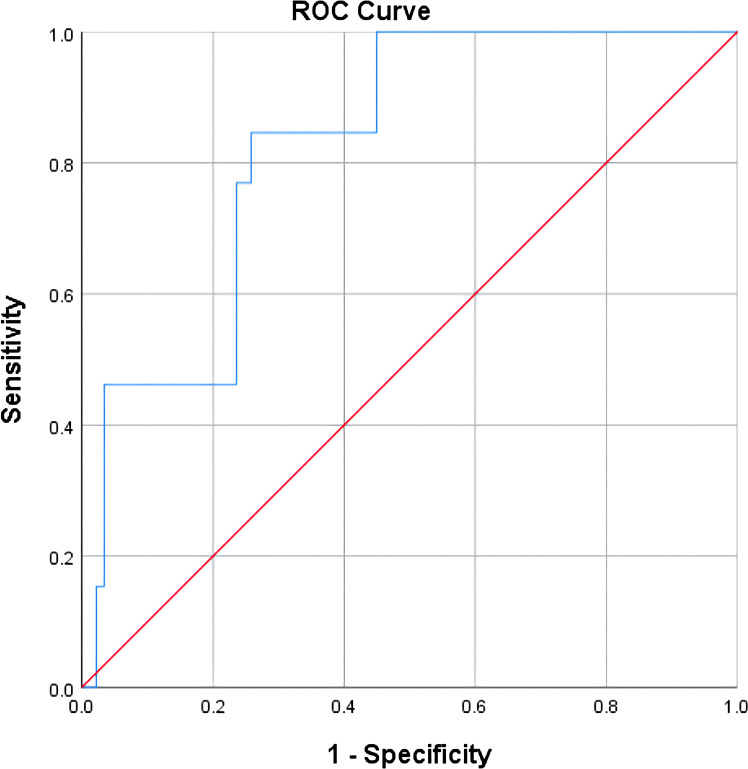
Predictive value of combined assays for the efficacy of neoadjuvant chemotherapy in patients with advanced ovarian cancer.

The test results of NLR, CA125, CA199, CEA, CA153, and HE4 were used to establish a ROC curve for predicting the ineffectiveness of NACT, and it was found that CA125 and HE4 were significant in predicting the efficacy of NACT (*P*<.05; **Table 8**). Including both variables in the logistic regression analysis, the ROC curve showed an area under the curve of 0.825, sensitivity of 84.6%, and specificity of 74.2% for the combined CA125 and HE4 assays to predict the inefficacy of NACT in patients with advanced ovarian cancer (**Figure 8**; **Table 9**).

**Table 8:** ROC curve analysis of the factors predicting the failure of neoadjuvant chemotherapy.

Variable	AUC	Standard error	P	95%CI
NLR	0.465	0.109	0.681	0.25~0.679
CA125	0.32	0.068	0.036	0.186~0.454
CA199	0.488	0.085	0.892	0.322~0.654
CEA	0.43	0.064	0.413	0.305~0.555
CA153	0.469	0.065	0.722	0.343~0.596
HE4	0.183	0.05	<0.01	0.086~0.28

NLR, neutrophil-to-lymphocyte ratio; CA125, cancer antigen 125; CA199, carbohydrate antigen 19-9; CEA, carcinoembryonic antigen; CA153, cancer antigen 153; HE4, human epididymis protein 4.

**Table 9:** Joint inspection Logistics regression analysis.

Parameter	B	SE	WaId	df	Sig	OR
CA125	-0.002	0.001	2.598	1	0.107	0.998
HE4	-0.013	0.005	5.361	1	0.021	0.987
Constant	10.177	3.088	10.864	1	0.001	26296.803

CA125, cancer antigen 125; HE4, human epididymis protein 4.

## Discussion

Ovarian cancer typically presents insidiously and is difficult to detect in its early stages, with nearly 70% of patients being diagnosed at an advanced stage (4, 5). Numerous investigations have demonstrated that surgical thoroughness significantly correlates with survival rates in patients with advanced ovarian cancer. Achieving satisfactory tumor reduction during PDS is a prerequisite for successful postoperative maintenance therapy. Moreover, attaining R0 resection is linked to a considerable enhancement in overall survival for those facing advanced stages of the disease (25–27). Patients facing advanced ovarian cancer often have metastases to multiple sites such as the peritoneum, greater omentum, intestines, liver, spleen, diaphragm, and lymph nodes. This multifocal spread complicates the direct application of PDS and significantly increases the risk of perioperative complications and mortality (27–29). NACT-IDS can increase the feasibility of minimally invasive surgery, potentially improving tumor reduction and reducing risks of postoperative complications and perioperative mortality. However, this approach might not extend postoperative survival, and could increase the risks associated with chemotherapeutic drug resistance and tumor recurrence (14, 15, 30–32).

The choice between PDS and NACT-IDS for patients with advanced ovarian cancer necessitates a careful evaluation and requires a multidisciplinary approach, incorporating insights from gynecological oncologists, pathologists, and the analysis of clinical indicators and imaging results. This collaborative effort aims to provide tailored and precise treatment to achieve the best therapeutic outcome (33, 34). Predicting the efficacy of NACT represents a crucial clinical inquiry for future research endeavors.

Research has reported a connection between chronic inflammation and the progression of ovarian cancer, highlighting the critical role of the inflammatory response in tumor proliferation, invasion, and migration by upregulating inflammation to promote tumor angiogenesis and inhibit antitumor activity (35, 36). Neutrophils, produced in the bone marrow, possess phagocytic, chemotactic, and bactericidal effects. They can also synthesize vascular endothelial growth factor (VEGF), which promotes abnormal neovascularization and accelerates the growth and metastasis of malignant tumor cells (37, 38). Lymphocytes, produced in lymphoid organs, are crucial for immune recognition and constitute the main cellular components of the immune response. A decrease in lymphocyte counts can diminish immune-mediated tumor antagonism, thereby facilitating the proliferation and metastasis of malignant tumor cells (39, 40). NLR is a leukocyte-associated inflammatory marker suggestive of the body’s immune homeostasis. An increased NLR disrupts the immune homeostasis, resulting in an imbalance between antitumor immune response and protumor inflammatory response, which promotes tumor cell growth and metastasis, and bears a strong correlation with the progression and prognosis of ovarian cancer (16, 17, 41, 42).

CA125 is currently the most commonly used adjuvant indicator for the diagnosis, efficacy observation, and prognostic assessment of ovarian cancer, and kinetic changes for the prediction of satisfactory subtractive tumors have been widely studied (18, 19, 43, 44). Normalization of pre-IDS CA125 levels is an accurate predictor of satisfactory tumor-reducing surgery and is significantly associated with better survival rates. However, the CALYPSO and ICON-8 studies demonstrated that CA125 levels do not accurately assess patient sensitivity to chemotherapy (45–47). CA199 is a tumor marker associated with pancreatic, gallbladder, gastric, and colon cancers. In patients with ovarian cancer, CA199 is highly expressed and can be shed in large quantities from the cell membranes of the ovarian cancer cells into the bloodstream, showing remarkable sensitivity for ovarian cancer detection (20, 48, 49). CEA, a soluble glycoprotein with complex structure and embryonic antigenic properties, is mostly found in the fetal liver and gastrointestinal tract during the embryonic period, and is present in minimal quantities in tissues. Abnormally elevated levels of CEA may affect the differentiation of tumors and increase the risk of tumor infiltration and metastasis, which in turn affects the clinical regression of patients with ovarian cancer (21, 50, 51). CA153 was first found in breast cancer epithelial cells and can cause changes in cell surface glycans due to activation of glycosyltransferases during carcinogenesis. This marker is present in a variety of adenocarcinomas and has increasingly been used as an adjunctive indicator for the diagnosis and differentiation of ovarian cancer in recent years (22, 52, 53). HE4 is an acidic inhibitory protein that exacerbates ovarian cancer by facilitating early infiltration and metastasis, thereby accelerating the disease’s progression. Elevated expression levels of HE4 may signal poor clinical regression in patients with ovarian cancer, reflecting the disease’s progression and prognosis to a certain extent (19, 23, 48, 54).

In this study, NACT demonstrated effectiveness when the HE4 level exceeded 422.95 pmol/L. NLR exhibited a remarkable sensitivity of 93.3% in predicting the effectiveness of NACT in individuals facing advanced ovarian cancer, while CA199 displayed a high specificity of 92.3% in the same regard. In individuals facing advanced ovarian cancer, HE4 was the best predictor of NACT efficacy, boasting a sensitivity of 75.3% and a specificity of 84.6%, whereas CEA and CA153 were poor predictors of NACT efficacy in these patients, displaying low sensitivity and specificity. The combined HE4 and CA125 test exhibited an area under the curve of 0.825 for predicting the ineffectiveness of NACT in individuals facing advanced ovarian cancer, demonstrating a sensitivity of 84.6% and a specificity of 74.2%. Among the indicators of NLR, CA125, CA199, CEA, CA153, and HE4 in advanced ovarian cancer, the single tests had some degree of defects, thus affecting their value, whereas the combined tests made up for each other’s defects, thereby improving the prediction of the effectiveness of NACT in individuals facing advanced ovarian cancer and accurately selecting the patients suitable for NACT, avoiding unnecessary excessive NACT.

This study does have some limitations. First, the investigation was a prospective clinical study with participants recruited from the same hospital, and no multicenter study was conducted, thereby resulting in geographical differences. Second, in this study, the distribution of participants included 54 patients at stage IIIC, 36 at stage IVB, and 12 at stage IVA, while stage IIIA and stage IIIB had no representation, and therefore, the results of the study may be biased. Third, the sample size was insufficient. Collaboration with other centers is required to reduce the incidence of error bias. Fourth, the use of cisplatin instead of carboplatin in the chemotherapy regimen may have increased nephrotoxicity, potentially affecting efficacy. Fifth, the relationship between types of pathology and chemotherapy efficacy was not considered, and mixed histopathology types may have biased the results. Certain types of pathology do not respond to NACT, and analysis of high-grade serous adenocarcinoma alone may be more clinically relevant. Sixth, the study was limited to a single chemotherapy regimen. The potential for combining polymerase (PARP) inhibitors with chemotherapy, antiangiogenic therapy, immune checkpoint inhibitors, and other DNA damage response modifying drugs for NACT deserves further exploration (6). NLR, CA125, CA199, and HE4 dynamic testing can provide valuable insights into forecasting the effectiveness of NACT for individuals facing advanced ovarian cancer, and in our future clinical works, we should continue to improve our surgical skills and apply a variety of new technologies (55, 56), so that more patients can achieve a true R0 resection by receiving individualized and precise treatment plans, thereby improving their quality of life and maximizing therapeutic outcomes.

## Conclusion

Ovarian cancer, often diagnosed at advanced stages due to its insidious early symptoms, remains the leading cause of death from gynecological malignancies. The predictive accuracy of NACT’s efficacy in advanced ovarian cancer is significantly enhanced by NLR’s high sensitivity and CA199’s high specificity. Furthermore, the synergistic analysis of CA125 and HE4 demonstrates an improved predictive effect, which can accurately select patients suitable for NACT, determine the appropriate timing of the IDS surgery, achieve a satisfactory effect of tumor reduction, avoid unnecessary excessive NACT, reduce the occurrence of perioperative complications, reduce the postoperative chemotherapy resistance and toxic side effects, and carry out the individualized precision treatment to achieve the best therapeutic effect. To further improve patient outcomes, ongoing enhancement of surgical techniques and integration of innovative technologies are essential. These advancements enable more patients to achieve a true R0 resection, providing a solid foundation for subsequent chemotherapy, targeted therapies, and immunotherapy, ultimately elevating survival rates and quality of life for individuals facing advanced ovarian cancer.

## Data availability statement

The raw data supporting the conclusions of this article will be made available by the authors, without undue reservation.

## Ethics statement

The studies involving humans were approved by Ethical Committee of Ganzhou Cancer Hospital. The studies were conducted in accordance with the local legislation and institutional requirements. The participants provided their written informed consent to participate in this study.

## Author contributions

JH: Writing – review & editing, Writing – original draft, Project administration, Conceptualization. DD: Writing – original draft, Investigation, Data curation. HC: Writing – original draft, Investigation. DL: Writing – original draft, Investigation. QW: Writing – original draft, Investigation. CL: Writing – original draft, Data curation. YL: Writing – original draft, Investigation, Data curation. YY: Writing – original draft, Conceptualization.
